# “Industrial citizenship” and social inequality in Japan: the dynamics of contract and status in shaping inequalities

**DOI:** 10.3389/fsoc.2025.1647338

**Published:** 2025-09-24

**Authors:** Jun Imai

**Affiliations:** Department of Sociology, Faculty of Human Sciences, Sophia University, Tokyo, Japan

**Keywords:** industrial citizenship, labor and employment relations, Japan, deregulation and re-regulation, diversification of employment, class analysis, contract and status

## Abstract

This paper reinterprets social inequality in Japan through the concept of industrial citizenship—a framework that understands inequality not as the result of structurally and economically determined class positions, but as the historical product of contestations over citizenship. These struggles, embedded in labor relations, intertwine the logics of contract and status, shaping context-specific employment relations, including rights and obligations for different categories of workers. Rather than assuming the universality of class, this approach highlights how institutionalized struggles over inclusion and recognition produce divergent hierarchies. In postwar Japan, industrial citizenship developed into *company citizenship*, where regular employment status was confined within the organizational boundaries of individual firms. This model generated inequality structured not by class, but by company size, gender, and employment status. As employer prerogatives were consolidated, norms of inclusion—based on company membership and flexible abilities—became institutionalized and deeply embedded. Even after neoliberal reforms that ostensibly emphasized contractual arrangements, the underlying logic of company citizenship persisted. Legal changes clarified the boundaries between employment statuses, while new employment tracks further stratified regular employees—both outcomes rooted in the logic of company citizenship. Crucially, these arrangements were sustained not only by managerial authority but also by worker consent shaped by company citizenship norms, making inequality appear fair and thus institutionally stable. By foregrounding industrial citizenship, this paper offers an alternative to class-centered frameworks. It emphasizes how historically contingent configurations of status and contract shape the (reproduction of) inequality, providing a comparative tool for analyzing stratification in capitalist democracies beyond liberal assumptions.

## 1 Introduction

Placing Japanese patterns of social inequality within the framework of international comparison presents particular challenges. In most cross-national analyses, the concept of “class” remains the dominant analytical tool. However, the explanatory power of traditional class-based frameworks—such as Goldthorpe’s class schema, which presumes economically rational actors—has proven relatively limited in the Japanese context (Sato, 2008a, 2008b). Empirical studies in stratification research consistently indicate that patterns of inequality in Japan are more effectively explained by status-related factors such as company size—which reflects the inter-firm hierarchy in Japan—gender, and employment status^1^. These observations have led some scholars to characterize Japan as an “exception,” a society in which feudalistic status distinctions remain salient despite its modern capitalist-democratic institutional structure, akin to other Western societies. Rather than viewing this characterization as an anomaly, this paper argues that such patterns point to the limited applicability of conventional class categories. Rather than relying on class typologies based solely on the development of capitalism, this analysis incorporates another institutional feature common to capitalist democracies—the development of citizenship—and advances the concept of industrial citizenship, as originally formulated by T. H. Marshall and subsequently elaborated by scholars such as Giddens and Streeck.

This framework reconceptualizes class dynamics as processes through which labor movements incorporate inherited status norms and logics into the institutional formation of labor relations. This approach shares its perspective with those who emphasize labor relations and their power dynamics—particularly the weakness of labor in Japan—in explaining inequality (i.e., Imai, 2011a; Thelen, 2014; Watanabe, 2018; Shibata, 2020). Yet it differs by stressing not only power relations but also their normative underpinnings. Industrial citizenship thus provides a more effective lens for understanding the emergence of socially specific categories and hierarchies among workers. What appears as “class” in the British context may be seen as one historically contingent form of this process; in other societies, divergent patterns of stratification emerge from the same institutional tensions. In Japan, the development of industrial citizenship—realized in the form of “company citizenship”—has produced a stratification pattern shaped more by company size, gender, and employment status than by conventional class boundaries. This framework is also effective in accounting for both the persistent inequality between regular and non-regular workers, as well as the growing differentiation within regular employment in Japan, by focusing on status norms.

This paper begins by introducing the concept of industrial citizenship and tracing its historical development and defining characteristics within the Japanese context, with particular attention to the conflicts and negotiations in labor relations that are commonly framed as class dynamics. It then demonstrates how the reforms of labor market and employment relations over the past three decades have remained deeply embedded in the institutional logic of company citizenship, a Japanese version of industrial citizenship. It proposes a more nuanced framework that incorporates politically and culturally embedded understandings of social inequalities. The following sections revise and adapt materials from Imai (2011a, 2011b) and Imai (2015), while also incorporating translated and further developed content from Imai (2021)^2^.

## 2 Contract and status in the studies of social inequalities

### 2.1 From status to contract: in theory and in reality

**Figure 1:**
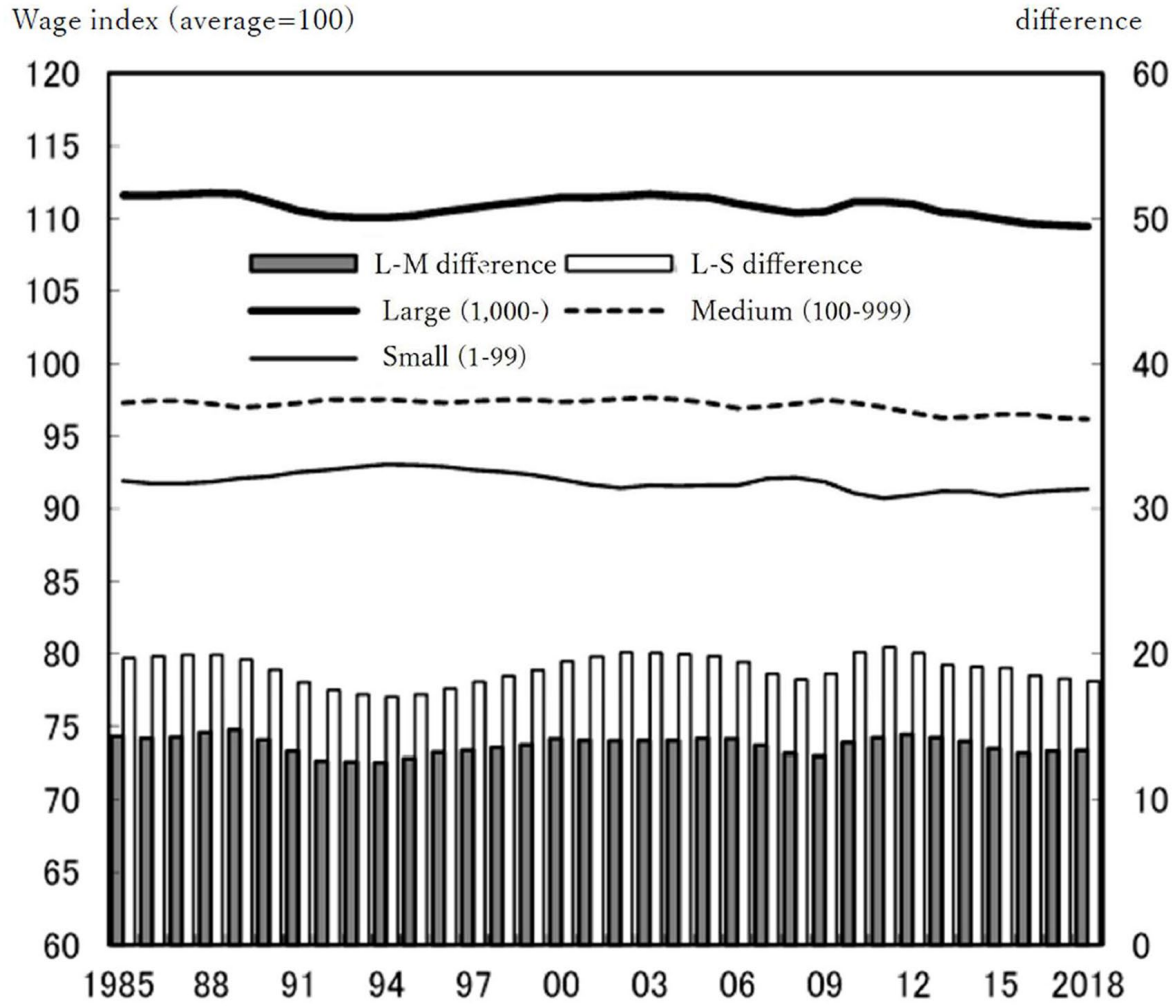
Wage differences by company size: 1985–2018. Source: Rengo Wage Report (Rengo, 2019, p. 5, translated by author).

The empirical realities of Japanese society continue to underscore the relevance of concepts more closely tied to *status* than to *class*—despite Japan being a prominent example of a capitalist democracy. The disparity in employment conditions by firm size has remained largely unchanged from 1985 to 2018—even when considering wages alone, let alone other welfare measures (Rengo, 2019, see Figure 1). Rengo has reported only a slight narrowing of disparities over the past five years (Rengo, 2023).

**Figure 2:**
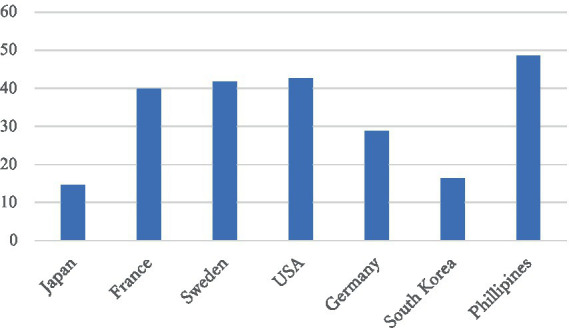
Women in management, international comparison (%). Source: White Paper on Gender Equality (Cabinet Office, 2024, p. 123).

While women’s labor market participation has increased and the gender wage gap among regular employees has shown signs of narrowing, women continue to face significant structural disadvantages. As of 2023, only 14.6% of managerial positions were held by women—a figure that is exceptionally low in international comparison (Cabinet Office, 2024, p. 122–123, see Figure 2).

Non-regular employment has expanded significantly over the past three decades, now accounting for approximately 35% of all employed workers in Japan. However, the gender disparity is substantial: more than 50% of employed women hold non-regular positions, compared to just over 20% of men. Estimates suggest that individuals who move from regular to non-regular employment experience a substantial decline in hourly wages (Nagase, 2018), underscoring the importance of employment status over individual human capital. Non-regular workers in Japan face not only wage disadvantages and employment instability but are also frequently excluded from corporate welfare and, in some cases, even from basic social entitlements such as public health insurance and pension coverage—rights that are ostensibly guaranteed to all citizens. Non-regular employment intersects sharply with gender and generational divides, producing the labor market segmentation (Lechevalier, 2015), meaning the difficulty of transitioning into regular employment that often has long-term consequences for individuals’ career trajectories (Iwakami, 2015; Sato, 2021). Japan’s employment relations continue to reproduce entrenched status-based inequalities, prompting some scholars to describe it as a ‘*kaisha mibun-sei*’ (Nomura, 2007) or a ‘*koyō mibun shakai*’ (Morioka, 2015). While *mibun* is often translated as “status,” it more accurately denotes the enduring presence of institutional logics reminiscent of feudalism within contemporary labor relations—a perspective that tends to align with narratives of Japanese exceptionalism.

What is striking here is the persistence of status-based inequality even after more than three decades of neoliberal reform in Japan—reforms that have emphasized free competition, individualism, and autonomy across multiple dimensions, including public policy, corporate labor management, and individual work-life orientations. Given the centrality of the individualized contract in neoliberal logic, one might expect that disparities would increasingly be explained only by differences in personal attributes such as human capital. In principle, inequality not attributable to individual merit should have diminished. However, this has not been the case. A plausible explanation is that Japanese employment relations continue to incorporate status elements. While employment relations are formally grounded in labor contracts—and thus aligned with the individualistic social relations characteristic of modernity—they remain significantly shaped by status-based norms.

The ascendancy of contract over status was viewed by social theorists as a foundational transformation marking the transition from premodern to modern societies. Legal theorist Henry Maine famously described this trajectory as a shift “from status to contract” (Maine, 2013). This transformation entailed the emergence and proliferation of contractual relationships, signaling a broader societal move away from decision-making grounded in familial or communal authority toward one centered on the individual as the principal bearer of rights and agency (Bendix, 1981). The breakdown of feudal social order, the rise of meritocratic social mobility over hereditary privilege, and the replacement of status-based social closure with individual-based exploitation via market mechanisms are all interpreted as key aspects of this shift (Turner, 1988, p. 23). The phrase “from status to contract” thus signifies a societal transformation in which positions once determined by status are increasingly determined by contract. As such, structures of social inequality are also expected to reflect this transformation.

Class analysis, a central tradition in the study of social inequality, largely incorporates this transformation within its theoretical framework. Karl Marx himself, despite his critique of capitalism, retained the liberal understanding of contract as a voluntary and equal exchange, a perspective also held by classical economists (Marx, 1969, Vol. I: 306)^3^. He theorized that such contractual relations, under capitalist production, inevitably give rise to class relations between capitalists and wage laborers, under which the former systematically exploit the latter. Likewise, Goldthorpe, who formulated one of the most influential contemporary class schemas, anchors his argument in a theory of rational action grounded in transaction cost economics. His framework presupposes the existence of a liberal labor market—a social order based on individualistic and reciprocal contract relations—and constructs class schema accordingly (Goldthorpe, 2007, p. 108). Focusing particularly on the employer’s perspective in employment contracting, he conceptualizes the employment relationship as a principal-agent problem, wherein the objective is to induce workers to exert sufficient effort and accept managerial authority.

Goldthorpe’s schema, with its focus on contractual positions, stands in stark contrast to the Japan exceptionalist approach, which reduces inequality to enduring status structures. The societal model presupposed in his work is that of Britain (Hara and Seiyama, 1999, p. 11), which comparative institutional research often classifies as a prototypical liberal market economy (Hall and Soskice, 2001). Within this context, the class distinctions he draws are presumed to align with actual divisions embedded in employment management practices. However, such alignment may not extend beyond the British case. In Japan, for instance, although blue-collar and white-collar workers can be differentiated in terms of job content, this divide does not function as a primary axis of social inequality. As will be discussed in the next section, these groups have historically been subject to largely similar employment management systems. Thus, the distinctions central to Goldthorpe’s framework—such as between service and labor contracts—have not been institutionally significant in Japan, nor have they served as foundations for social inequality. When class analysis encounters cross-national differences that its schema cannot fully accommodate, it often supplements its explanatory capacity by appealing to differences in industrial structure or unique societal conditions (DiPrete, 2005, 2007; Sato, 2008a, 2008b). However, employment management systems are themselves part of the social organization that enables labor contracts to function, and must be examined as domains where status-based norms shape inequalities in ways specific to each society.

How, then, can we adequately recognize and explain the diversity of inequality structures (and mobility patterns) observed across different societies? A crucial starting point lies in recognizing that the dichotomy between *status* and *contract* does not sufficiently capture the complexity of historical reality. The relationship between status and contract is not one of mutual exclusivity or linear succession; rather, the two are deeply interrelated and intertwined, together shaping the fabric of social reality. The significance of Durkheim’s departure from his initial dichotomy of mechanical and organic solidarity—originally intended to distinguish between pre-industrial and industrial social relations—should not be overlooked. He argued that even contractual relationships rest upon a “non-contractual basis,” underscoring the normative and moral foundations necessary for the functioning of modern contractual social order. Although the modern era has positioned contractual relationships in opposition to status-based arrangements, the very conditions that make contracts operable may in fact be underpinned by traditional status elements. These two principles remain integral to the maintenance of social order, though their relative balance and concrete expressions vary across historical and cultural contexts (Durkheim, 2005, p. 196–224; Streeck, 1992, p. 43–45; Deakin and Wilkinson, 2005, p. 41–109).

The structure of social inequality can thus be understood as one of many possible social orders, produced through the complex interplay between the principles of status and contract. However, these two principles are not equally weighted in modern societies. The normative ideal tends to privilege contract, and employment relations are fundamentally organized around contractual arrangements. In most democratic capitalist societies, the explicit privileging of ascribed characteristics or the imposition of rigid barriers to mobility—core features of status-based arrangements—is widely regarded as illegitimate. Given this normative stance, a critical question arises: why and how do elements of status continue to be embedded in systems of inequality in ways that retain social legitimacy?

### 2.2 Contract by status – industrial citizenship via class dynamics

In industrialized democratic societies, employment relations, though potentially institutionalized along individualistic and market-oriented lines, have incorporated status elements, generating distinctive patterns of social inequality. This development is best understood through the concept of industrial citizenship, originally proposed by T. H. Marshall (1992). Marshall conceptualized industrial citizenship as a normative framework of rights and obligations that defines the social status of workers through ongoing negotiations among labor, employers, and the state. He positioned it as a secondary form of citizenship specific to industrial society (p. 26, 40–41). Marshall argued that workers in industrial society are granted particular rights linked to their respective status, accompanied by normative expectations regarding appropriate attitudes and conduct as employees. The hierarchical structure of these statuses reflects the political and cultural negotiations necessary to sustain contractual relations—relations in which status norms become embedded. This historical process did not unfold as a linear transition from status to contract, but rather as a complex dynamic in which status-based norms were interwoven into the institutional foundations of employment.

Marshall defines the concept of “status” in modern society through the historical development of citizenship rights, encompassing civil rights established in the 18th and 19th centuries, political rights, and the social rights that came to characterize the 20th century (p. 17). He argued that citizenship functions as a countervailing force to the dynamic rise of capitalism—a system inherently structured by inequality—and thus operates as a normative logic for mitigating social disparities (p. 18). However, Marshall also recognized that these two logics—citizenship and capitalism—are not simply oppositional, but deeply entangled in complex ways. One such entanglement is evident in the relationship between civil rights and the market economy. Civil rights, which Marshall identified as the first set of citizenship rights, guarantee individual liberties such as “freedom of speech, thought and faith, the right to own property and to conclude valid contracts, and the right to justice” (p. 8). These rights were indispensable for the formation of the economically independent individual and aligned closely with the emergence of a competitive market economy, particularly in the early stages of capitalism (p. 20). Yet, this historical trajectory also gave rise to movements aimed at regulating and counterbalancing the unrestrained expansion of the market economy—a process famously described by Polanyi (2001) as the “double movement.”

The citizenship movements of the twentieth century were characterized by the use of collective civil rights, marking a shift in agency from the individual back to the collective (Marshall, 1992, p. 25). Marshall first observed that workers became able to organize labor unions due to the earlier establishment of civil rights. He then emphasized how collective actions by labor unions to defend and expand citizenship significantly influenced wage levels, living standards, and the realization of social rights. From the mid-twentieth century onward, even employers and the state could no longer ignore such demands and mobilizations. What had once been “crude economic bargaining” came to resemble “a joint discussion of policy” (p. 41). These negotiations among labor, employers, and the state helped to improve working conditions and situated employment more clearly within the framework of the welfare state. The development of institutional links between employment and welfare established a normative framework of rights and duties—industrial citizenship—which served to stabilize the social status of wage labor.

Although the notion of citizenship is often assumed to imply *a priori* equality among citizens, Marshall pointed out that the normative framework of industrial citizenship actually functions as a status-bearing principle that can generate inequality. He stressed that obligations in modern society may derive not only from contract but also from status, and that there are numerous precedents in which contracts are subordinated to status (p. 41). This insight highlights the constitutive role of status norms in the establishment of contractual relations. Marshall further illustrated this point by examining how “fair wages” are socially determined through reference to the notion of status.

For example, he asked:

*What ought a medical specialist or a dentist to earn, we ask? Would twice the salary of a university professor be about right, or is that not enough? And of course, the system envisaged is one of stratified, not uniform, status. The claim is not merely for a basic living wage with such* var*iations about that level as can be extracted by each grade from the conditions in the market at the moment. The claims of status are to a hierarchical wage structure, each level of which represents a social right and not merely a market value. Collective bargaining must involve, even in its elementary forms, the classification of workers into groups, or grades, within which minor occupational differences are ignored* (p. 42).

This observation is critical to understanding how labor contracts are constituted. Even wages—the employment condition seemingly most susceptible to market mechanisms—are not determined through competitive exchange among utility-maximizing individuals, but are instead shaped by status perceptions, particularly those rooted in traditional group-based classifications and hierarchies. Marshall thus observed that collective struggles over citizenship—most notably by organized labor—help institutionalize status hierarchies among worker categories, including differentiated entitlements to wages and social rights. These hierarchies become embedded within a society ostensibly based on individualistic and meritocratic contracts (p. 39–40).

What is revealed here is that the rights of citizenship, once guaranteed to individuals as legal equals, serve as a medium for conflict between labor and employers. These struggles not only improve the conditions of employment but also intersect with the state’s interests and interventions, thereby facilitating the expansion of social rights (Giddens, 1982, p. 174; Streeck, 1992, p. 52). At the same time, negotiations among labor, employers, and the state weave status logics into contracts, making the employment relationship itself contingent upon those logics. The result is the formation of a stratified system of social positions, defined by rights and duties, that constitutes industrial citizenship (Streeck, 1992; Dukes and Streeck, 2023). Although the status logics embedded in these positions may bear traces of pre-modern (or “feudal”) elements, the resulting structures of inequality become socially legitimate through the very process of negotiation under the framework of modern citizenship. In this sense, they acquire a durable and stabilizing character. As Marshall himself put it, “the two [citizenship and social class] are still compatible, so much so that citizenship has become, in certain respect, the architect of legitimate social inequality” (Marshall, 1992, p. 7).

A further analytical advantage of the concept of citizenship lies in its capacity to illuminate both the processes of inclusion and exclusion. While citizenship is often associated with egalitarian ideals, its historical and theoretical foundations suggest otherwise: it functions as a mechanism that privileges certain actors based on national belonging or perceived civic competence (Turner, 1993; Tilly, 1998; Glenn, 2000). Rather than inherently guaranteeing equal rights, citizenship delineates boundaries between members and non-members, legitimizing differentiated entitlements.

Industrial citizenship also operates within this logic of selective inclusion. As a secondary form of citizenship specific to the domain of employment and welfare, it is not universally granted but must be negotiated through institutionalized relationships among workers, employers, and the state. The rights and entitlements associated with industrial citizenship are acquired through collective mobilization—most visibly through union activity—and thus depend on organizational membership and bargaining capacity. These mobilizations generate normative frameworks for fairness and justice, but these are not universalistic. The boundaries of equality are defined by the shared conditions and identities of those engaged in the bargaining process. Labor unions, for example, articulate standards of fair compensation and entitlements for their members, but in doing so, they exclude non-members from these gains. The logic of fairness, therefore, is grounded in group-specific interests rather than universalist principles. In this way, industrial citizenship simultaneously institutionalizes mechanisms of inclusion and reproduces durable forms of exclusion.

### 2.3 Summary

The concept of class warrants reconsideration, particularly in light of its limited explanatory power in the Japanese context. Rather than centering analysis solely on the processes of capitalist industrialization and the logic of economic rationality, greater attention should be directed toward the emergence of civil society and the role of status recognition embedded in specific socio-historical contexts.

This paper proposes the concept of *industrial citizenship* as an alternative analytical lens. Industrial citizenship refers to a set of status rights and obligations that arise primarily within the employer-employee relationship—a relationship typically characterized by asymmetrical power (Marshall, 1992; Streeck, 1992) and patterned differently across local political contexts (Crouch, 1993; Somers, 1993, p. 595). It tends to develop within institutional configurations in which employment relations are closely interwoven with welfare provision—an interdependence often described as the *employment–welfare nexus* (Rubery, 2010; Ebbinghaus and Manow, 2001). It is essential to examine how industrial citizenship is historically shaped by political and cultural negotiations among the state, employers, and workers (Fligstein, 2001). The content of industrial citizenship reflects this historical trajectory and institutionalizes a specific logic of fairness and equality that these actors – especially workers – bring into negotiation. However, once institutionalized, industrial citizenship may also legitimize forms of social inequality and exclusion, insofar as they are reproduced through the very institutional arrangements that confer negotiated entitlements (Marshall, 1992).

The next section demonstrates how the Japanese variant of industrial citizenship – *company citizenship* – has been historically constructed and delineates its defining characteristics.

## 3 Company citizenship – how inequalities in Japan is shaped by the dynamics of industrial citizenship

In Japan, industrial citizenship has been constructed as a form of “company citizenship,” institutionally embedded in the status of regular employment, which is segmented and confined within each company organizations (Imai, 2011b, 2015, 2021; Gordon, 1993). The key entitlements associated with this status include employment security, life-stage-adjusted wages, and corporate welfare benefits—provisions that enable workers (and their families) to envision stable life trajectories. Access to these rights is conditioned on workers’ strong organizational commitment and their ability to meet the demands for flexibility, requiring a particular disposition and lifestyle often symbolized by the figure of the “company man” (Kumazawa, 1997; Imai, 2011b).

Company citizenship was historically shaped through a specific configuration of labor relations, often described as a “productivity coalition” between employers and increasingly cooperative unions and workers, supported by state welfare and tax policies. This process of negotiation unfolded at multiple levels—societal, organizational, and workplace. Within a legislative environment that constrained contractual diversity and external labor mobility throughout most of the postwar period until the 1980s, the shift in power dynamics—from strong labor participation to the predominance of managerial authority—defined the status of regular employment through the codified rights and duties of company citizenship. Over time, company citizenship—as the institutional logic underpinning Japan’s employment–welfare nexus—came to privilege male regular employees at large firms over women and workers in smaller enterprises, institutionalizing a negotiated order of social inequality.

This section first provides a historical account of how these status rights and obligations were constituted and how they came to embody a normative sense of equality and fairness within the institutional development of the employment–welfare nexus. The following sections examine how the logic of company citizenship continues to shape the cognitive frameworks of key organizational actors.

### 3.1 Early development of company citizenship

Postwar legislation concerning employment relations established a social space in which the status of regular employment could develop. Influenced largely by the Occupation authorities’ efforts to reform Japan into a “democratic” society, laws such as the Labor Standards Act (1947) and the Employment Security Law (1947) were enacted to eliminate feudal practices in the labor market (Gordon, 1993; Takanashi, 1993). These laws can be said to have restricted contractual diversity and external labor mobility: fixed-term contracts were generally limited to durations of less than one year, and labor market intermediation was prohibited except when conducted by the state, labor unions, or educational institutions. As this legal framework remained largely unchanged until the late 1980s, labor relations played a central role—and state social policy a more supportive one—in the construction of the status of the regular employee.

Labor unionism surged in the immediate postwar period within a social space structured around company- and workplace-based unions (Nimura, 1987; Gordon, 1993). Several explanations have been proposed for this organizational form: the absence of a guild tradition (Gordon, 1998, p. 29–30); the organizational logic of daily face-to-face interactions at the workplace level (Nimura, 1987, p. 82–86); and the potential institutional legacy of the Industrial Patriotic Societies (*sangyō hōkokukai*), state-sponsored wartime organizations established to mobilize workers in support of Japan’s military objectives (Nitta, 2008, p. 207). Although the momentum of labor unionism had stalled by the end of the 1940s—due in part to policy shifts aimed at reasserting managerial authority—the labor disputes of this period laid the foundation for key features of Japan’s postwar employment relations (Hyodo, 1997; Gordon, 1998). During this time, labor unions demanded participation in workplace decision-making, stable employment and wage security, and the elimination of discrimination against blue-collar workers. Over time, these demands were institutionalized in practices such as lifetime employment, seniority-based wage structures aligned with life stages, and the equalization of status among regular employees, which effectively erased the class divide—particularly the blue-collar/white-collar distinction that persisted as a key axis of social cleavage in many Western societies. These developments laid the institutional foundation for the enclosure of resources and opportunities within individual firms for those employed as regular workers, ultimately underpinning the emergence of a robust middle-class consumer culture in the subsequent decades (Imai and Sato, 2011).

Two inherent limitations were already embedded in the emerging framework of industrial citizenship as a principle of equality: it did not challenge the inequalities produced by corporate hierarchies—initially coordinated during the wartime to ensure necessary production and later reestablished by the Ministry of International Trade and Industry (MITI) to support the developmental, catch-up economy^4^—and it failed to address gender-based disparities. The first limitation was reinforced by the formation of enterprise unions. Unlike societies where occupational unions were institutionalized, Japan lacked a cross-organizational standard for working conditions. As a result, company-specific labor relations prevailed. This, in turn, allowed vertically integrated corporate hierarchies—initially shaped during the war and reorganized during the period of high economic growth—to become a major axis of social inequality.

The second limitation illustrates how the very definition of membership in industrial citizenship entailed mechanisms of inclusion and exclusion. A key component in this process was the life-stage adjusted wage, institutionalized as a form of the family wage. The family wage was inherently premised on the “male breadwinner” model, and its institutionalization in conjunction with male-breadwinner-oriented social security schemes significantly contributed to the exclusion of women from company citizenship.

For instance, the exclusion of women from regular employment status became increasingly evident during this period. This trend became especially pronounced after the Korean War^5^. Although the war facilitated Japan’s economic recovery from postwar devastation, the subsequent economic adjustment created pressure to reduce the workforce. This was achieved, in part, by “tapping married women on the shoulder,” as both employers and labor unions assumed they had access to their husbands’ income (Hisamoto, 1998, p. 107). This approach stands in stark contrast to the labor unions’ simultaneous efforts to organize male temporary workers, demanding their inclusion in the category of regular employees (Hisamoto, 1998). Such gendered labor practices played a central role in delineating members and non-members of the firm, effectively excluding women from regular employment status. However, this shadow cast by the postwar labor movement has often been overlooked or obscured by the optimistic narratives of economic and social progress associated with the rapid growth of the 1960s, which served to legitimize the employment institutions established in the immediate postwar period.

### 3.2 “Company man”: a symbol of company citizenship

During the period of rapid economic growth, a qualitative shift occurred in labor relations, marked by the formation of a “productivity coalition” in which labor unions were tamed and co-opted to cooperate in pursuit of increased firm productivity. Most notably, employers made significant efforts to replace militant “first unions” (*dai-ichi kumiai*) with more cooperative “second unions” (*dai-ni kumiai*) in order to reassert managerial authority throughout the 1950s and 1960s. To legitimize the second unions, employers introduced a range of corporate welfare programs—including company pensions, health insurance, retirement allowances, preferential savings schemes, company housing, and low-interest housing loans—many of which were supported by state social security and tax policies (Koike, 1977; Kumazawa, 1997). These programs proved highly effective in mobilizing workers’ commitment to corporate goals such as increased production and improved productivity. These corporate welfare programs provided cooperative workers with economic rewards, and in return, workers’ collaboration granted employers “a freer hand to introduce new technology, redefine jobs, rearrange work, and transfer employees” (Gordon, 1993, p. 384), thereby effectively reinforcing managerial authority.

It is important to note that these outcomes were made possible by social policies rooted in a developmentalist orientation, such as the introduction of preferential tax treatment aimed at supporting the more “productive” segments of the labor force (Manow, 2001; Shinkawa and Pempel, 1996; Kim, 2010). Health insurance, the pension system, and retirement allowances serve as illustrative examples of this policy orientation (Osawa, 1993). For instance, although Japan operates a universal pension system, in response to employers’ efforts during the 1950s and 1960s to promote long-term worker commitment through economic incentives, the pension system became increasingly segmented. Company-based pensions were granted preferential tax treatment that disproportionately favored larger firms (p. 190–195). This arrangement effectively created incentives for workers to seek and maintain employment in (especially large) firms, thereby contributing to the formation of what Osawa (1993) calls a “corporate-centered society,” in which access to livelihood security is primarily ensured through firm membership. These state policies not only reinforced labor market segmentation closely tied to welfare provision but also privileged the status of regular employment, particularly for those employed by large corporations.

Labor unions themselves began to shift their focus from workplace participation to negotiating economic redistribution, most notably through the institutionalization of *Shuntō* (the “spring offensive”)—a coordinated annual wage bargaining campaign that began in 1955. The *Shuntō* movement played a significant role in raising the average income of Japanese workers, and, together with expanding promotion opportunities driven by postwar economic growth, laid the groundwork for the emergence of a robust middle class in Japan. This shift in union strategy also gradually altered the meaning of “participation”: from involvement in decision-making to a focus on productivity, and from power sharing to profit sharing (Gordon, 1993). Especially following the oil crises of the 1970s, amid growing concerns over employment security, labor unions increasingly retreated from labor-management decision-making in order to safeguard jobs and maintain workers’ living standards. In this context, a more acquiescent attitude toward management’s demands for flexibility became normalized among regular employees. Workers came to accept management’s unilateral authority to evaluate individual performance, loyalty, and adaptability—what Kumazawa (1997, p. 40) refers to as “the ability to be flexible”—along with the growing institutionalization of inter-worker competition.

This shifting power balance in labor relations helped shape the distinctive set of expectations and obligations associated with regular employment, exemplified by the figure of the “company man” (*kaisha ningen*)—a worker who devotes his entire life to the company. The oil crises of the 1970s compelled Japanese firms to undertake restructuring in a belt-tightening manner, including the implementation of various labor-saving measures. The Plaza Accord of 1985 further accelerated this trend. These external economic pressures challenged corporate productivity and prompted significant changes in both labor union strategies and labor-management relations. They also reinforced the legitimacy of the already entrenched “principle of productivity,” which increasingly constrained wage increases secured through *Shuntō* negotiations (Kumazawa, 1993; Kume, 1998). Major national union federations—particularly those representing the private sector—began to refrain from demanding substantial pay raises, instead emphasizing employment security and the preservation of workers’ living standards (Kume, 1998). Together with labor market regulations and social policies that discouraged inter-firm mobility, this strategic shift further limited workers’ choices, making acquiescence to management’s demands for flexibility the only realistic option for many.

The relentless pursuit of greater efficiency manifested in various forms and expanded the duties associated with company citizenship. The widespread adoption of the just-in-time production system required Japanese workers to acquire multi-tasking capabilities (Kumazawa, 1997). Adjustments to working hours—particularly through overtime—became a key mechanism for coping with economic fluctuations; as Dore (1986) noted, reducing overtime was among the most readily implemented cost-cutting measures in Japanese firms. During this period, intra-firm (or group) labor adjustments also became institutionalized through practices such as *shukkō* (temporary transfers) and *tenseki* (permanent transfers), which enabled employers to manage workforce size by reallocating employees to subsidiary firms (Inagami, 2003). These practices were made possible by regular employees’ willingness to accept varied assignments, flexible schedules, rotational duties, and regional relocations—thus allowing firms to achieve numerical and functional flexibility in distinctive ways (Imai, 2011a). Motivated in part by the fear of becoming a social equal to workers in smaller firms and to women—both of whom were less privileged within, or largely excluded from, the privileges of company citizenship—management strategies aimed at securing total commitment proved highly effective, ultimately giving rise to the archetype of the “company man” (Gordon, 1993, 1998). Meeting these expectations became a prerequisite for being recognized as a full citizen within Japan’s corporate-centered society.

### 3.3 Summary

Throughout the historical period examined thus far, postwar cooperation between workers and employers—the” productivity coalition”—gave rise to a distinctive social status associated with regular employment. This status, characterized by a set of privileges and obligations, came to be defined as *company citizenship*. The emergence of this citizenship status was grounded in a power relationship in which the interests of workers were subordinated to those of employers. Specifically, workers were expected to subordinate their personal lives and family planning to the organizational needs of the firm and to demonstrate a committed disposition toward adapting to changing corporate environments. While these expectations may be described as requiring “the ability to be flexible” and further to be “company man,” they functioned as obligatory norms for organizational members. For example, compliance with job transfers was taken for granted; fulfilling such duties did not warrant special recognition, but failure to comply would result in negative evaluations.

The institutionalization of company citizenship served to legitimate patterns of inequality and exclusion based on firm size, gender, and employment status. In broader terms, postwar Japan functioned as an industrial society in which economic growth was a national goal, and workers employed by large firms—perceived as the most significant contributors to this goal—were effectively treated as first-class citizens. While employees of small and medium-sized enterprises (SMEs) may have gained access to equivalent rights if their firm’s financial capacity increased, in most cases they remained subject to similar power dynamics and behavioral expectations without receiving the same level of entitlements (Imai, 2021, p. 79–88). Likewise, women were incorporated into the workforce as second-tier industrial citizens, subordinated to male entitlements in a society where the male breadwinner ideology was deeply embedded in labor management, social security systems, and the tax regime. Gender-specific divisions of labor were legitimized and institutionalized, positioning women’s access to rights as derivative of men’s.

Although the preceding discussion has emphasized the association between a specific set of rights and obligations and the category of regular employment, the term *company citizenship*—rather than *regular employment citizenship*—is employed here for two key reasons. First, these rights and obligations are fundamentally grounded in firm-based membership. As shown in the historical analysis, many of these rights were negotiated at the level of enterprise unions organized within individual firms, rather than through occupation- or industry-wide union structures. To be sure, some entitlements—such as employment security—have become de facto national norms, and practices such as seniority wages have diffused widely within Japan’s labor management. This diffusion has given rise to a socially recognizable form of regular employment, allowing one to acknowledge that *company citizenship* includes a dimension of *regular employment citizenship*. However, this does not negate the fundamentally company-bound basis on which these entitlements are constructed.

Second, the content and level of entitlements associated with regular employment vary significantly across firms. Wages are strongly correlated with firm size, and other benefits—such as retirement allowances and pension services—are also linked to a company’s financial capacity (Shizume et al., 2021). As a result, considerable variation exists even within the category of regular employment. It is widely accepted that workers performing the same tasks may receive vastly different compensation depending on the company they work for, reflecting a landscape fundamentally at odds with the principle of equal pay for equal work. This inequality, structured by firm size, is rooted in Japan’s historical development of vertically integrated corporate groups (*keiretsu*), in the tax and social security policies that favor firm-based welfare provision, and in the broader institutional framework that privileges company-level arrangements. Accordingly, the concept of *company citizenship* more accurately captures the institutional logic underpinning labor rights and stratification in postwar Japan.

## 4 Recent developments shaped by “company citizenship”

The last thirty years have witnessed a significant reorganization of employment relations and labor markets in Japan. This section examines two key developments: the deregulation and re-regulation of labor markets, and the diversification of regular employment. Both cases demonstrate that these transformations—and the accompanying rise in social inequalities and exclusion—have been profoundly shaped by the declining significance of labor and the logic of company citizenship.

### 4.1 Deregulation of labor markets

In the 1980s, Japanese firms remained largely reliant on the flexibility of regular employees, supplemented by a relatively small segment of *paato* (part-time)^6^ workers drawn from local labor markets. However, by the late 1980s, increasing internationalization and structural changes in industry prompted firms to begin utilizing external labor forces in limited occupational areas. This shift provided the rationale for legislative developments such as revision of the Labor Standard Acts and the enactment of the Temporary Dispatched Work Law (TDW Law) in 1986. These are the first major changes of employment regulations since 1947. In line with the postwar tradition of policy-making, these were deliberated within tripartite advisory councils (*shingikai*) organized by the Ministry of Labor (MOL), comprising representatives of public interests (typically academics), employers, and labor unions. However, the deregulation efforts that began in the mid-1990s followed a markedly different negotiation process and led to a substantial expansion of non-regular employment.

The process of deregulation was spearheaded by employers and pro-deregulation advocates, whose influence became increasingly visible from the mid-1990s onward, amid the long-term decline in the political and institutional significance of labor (Imai, 2011a). Changes in the policy-making process and the composition of key actors can be summarized as follows: (1) agenda-setting functions were transferred from the traditional tripartite advisory councils to newly established deregulation committees; (2) these committees were placed under the jurisdiction of the Cabinet, excluded labor representation, and were gradually granted greater authority relative to ministry-based advisory councils—a trend that arguably peaked under the Koizumi administration (April 2001 – September 2006). These institutional changes strongly favored employer interests and enabled a diversification of labor commodification. Key developments included the expansion of fixed-term contracts (e.g., three- and five-year terms) across a range of occupations, the establishment and subsequent deregulation of the temporary dispatch work system, and deregulation of working time regulations.

**Figure 3:**
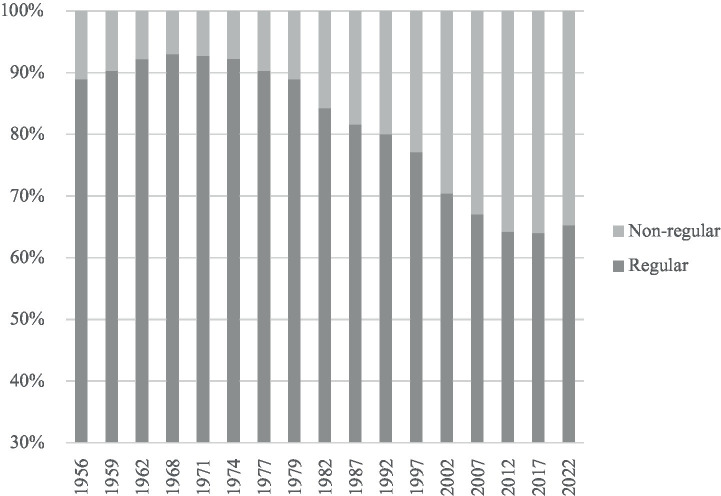
Increasing reliance on non-regular employment by Japanese employers, 1956–2022 (%). Source: Employment Status Survey, time series data (Statistics Bureau, 2025).

Deregulation provided firms with a range of new options for achieving labor flexibility, supplementing traditional internal mechanisms with the use of external labor markets. These reforms enabled employers to place differentiated expectations on newly expanded forms of employment (Imai, 2011a). Capitalizing on these institutional changes, Japanese firms significantly increased their reliance on non-regular employment (see Figure 3).

Newly created and/or expanded employment forms have emerged in ways that are disproportionately distributed by gender, occupation, and generation (Imai, 2011a; Osawa et al., 2013). For instance, as job areas traditionally occupied by women were increasingly externalized, over 50% of employed women are now classified as non-regular workers.

The logic of company citizenship has played a critical role in shaping the boundaries between regular and non-regular employment, deeply influencing the degree and structure of labor market segmentation. This logic was introduced into policy debates particularly from the side of labor. Labor unions, when granted a voice in the policy-making process, prioritized the protection of regular employees’ job security. While they sought to ensure that newly established or expanded forms of employment would not directly replace regular employment, they neglected the principle of equal treatment for non-regular workers. As a result, detailed analyses of labor market reforms indicate that non-regular employment statuses have been established outside the core of regular employment in terms of job content, contract duration, career mobility, and expected organizational commitment—all reflecting the embedded norms of company citizenship (Imai, 2011b). Deregulation thus gave rise to a social space characterized by various contract types and higher mobility, predominantly occupied by non-unionized and underrepresented workers who are consequently socially excluded.

**Figure 4:**
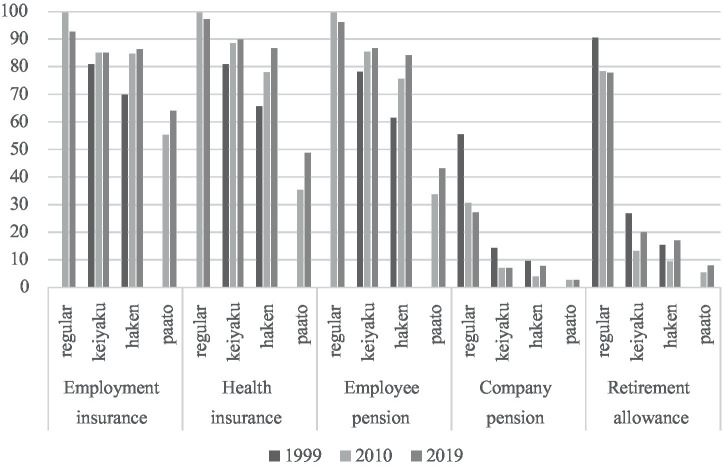
Enrollment rates in social insurance and corporate welfare schemes by employment forms (%). Source: Comprehensive Survey on the Employment Diversification (MOL, 2000; MHLW 2011, 2021).

This segmentation manifests in several key dimensions. First, non-regular workers are employed under contracts that offer significantly fewer protections and entitlements. Reflecting the power asymmetries embedded in the reform process, these employment forms have served as a new source of flexibility for employers, but not for workers (Imai, 2011a, 2011b). The mass dismissals in the aftermath of the Lehman Brothers collapse in the late 2008 illustrate this asymmetry particularly well: the majority of those affected were temporary dispatched workers, whose terminations were not legally classified as layoffs, but rather as the non-renewal or termination of dispatching contracts. Second, social exclusion has taken on a specific form, most clearly observed in the domain of corporate welfare (see Figure 4^7^).

Although some improvements have been made in terms of access to universal social security schemes—such as employment and health insurance and employee pension—significant disparities remain. Gaps are even wider in the realm of corporate welfare benefits, including company pensions and retirement allowances. While it should be noted that some of these benefits are increasingly seen as “luxuries” even among regular employees (such as company pension), substantial differences between employment statuses persist.

### 4.2 Re-regulation

The expanding inequality and social exclusion resulting from labor market deregulation gradually emerged as a salient social issue, gaining attention not only within academic discourse but also among the broader public. In response, three successive Liberal Democratic Party (LDP)-led administrations—Abe (Sep. 2006–Aug. 2007), Fukuda (Sep. 2007–Aug. 2008), and Aso (Sep. 2008–Sep. 2009)—acknowledged the need to address growing criticism of the outcomes of deregulatory reforms. The change in government in the summer of 2009, which brought an end to LDP rule, reflected increasing public awareness and demand for a shift in employment and labor market policies.

Three underlying shifts have gradually transformed social perceptions of non-regular employment and have shaped concrete interests in the re-regulation of labor markets. First, the growing number of non-regular workers has come to be viewed as a primary reason of rising inequality in Japan. Second, the demarcation between regular and non-regular employment has become increasingly blurred; *paato* workers are now frequently tasked with core functions that are indistinguishable from those performed by regular employees (Morozumi, 2008; Chin, 2008; Takeishi, 2002). Third, the number of workers supporting households on non-regular contracts has increased, reflecting a decline in the availability of full-fledged regular employment—traditionally allocated to male breadwinners (Chin, 2008).

While a favorable political climate and heightened public awareness appeared sufficient to prompt substantial policy shifts, the outcomes fell short of comprehensive re-regulation. During the early 2000s, two study groups were convened to examine the principle of “equal treatment,” which subsequently informed the amendments to the various labor related statues, including Part-time Work Law (hereafter, *Paato* Law). This section critically examines the extent to which these efforts failed to deliver transformative regulatory change, focusing on how the deliberations were shaped by prevailing logics of equality and fairness.

#### 4.2.1 Confirming standard employment centrism

A pivotal court ruling played a significant role in shifting public recognition of the principle of “equal treatment” between regular and non-regular employees. The case involved *Maruko Keihoki Co., Ltd.*, a horn manufacturer, where female *paato* workers filed a lawsuit claiming discriminatory treatment on the grounds that their work was comparable to that of regular employees. Notably, one of the plaintiffs had worked under a series of two-month contracts that had been consecutively renewed over a span of 25 years. Although classified as part-time workers, their daily working hours were only 15 min shorter than those of regular employees. In 1996, the Nagano District Court (Ueda Branch) delivered a landmark decision that, for the first time, recognized the principle of “equal pay for equal work” as applicable to the relationship between regular and non-regular workers. Given that this concept had not been widely institutionalized or operationalized in Japan, further deliberation was required to clarify its meaning and implications in practice.

In response to the court decision, the MOL convened a study group to address the issue. This group, officially titled the Study Group on Labor Management for Part-Time Work—commonly referred to as *Monosashi-ken*—comprised eleven members representing business, labor, and academia. The group released its final report in April 2000. In the executive summary, the stated aim was “to deliberate concrete and practical standards of equal treatment in various aspects of labor management that would facilitate the handling of such issues at the workplace level” (Monosashi-ken, 2000). Building on *Monosashi-ken*’s discussions, a subsequent body—the Study Group on Part-Time Work (*Paato-ken*)—published its report in July 2002. This second report aimed to translate the standards proposed by *Monosashi-ken* into statutory frameworks. These two reports are significant as they laid the foundation for subsequent legal and policy revisions, including changes to relevant labor laws and administrative guidelines.

Two study groups—*Monosashi-ken* and *Paato-ken*—proposed a dual framework for the application of equality principles, to be interpreted on a case-by-case basis. The first is *kintō shogū gensoku*, commonly translated as “the principle of equal treatment.” The second is *kinkō hairyo gimu*, or the “obligation to ensure balanced consideration” in treatment across different employment statuses.^8^ The former aligns with the European notion of “equal pay for equal work.” According to the reports, when the content of a non-regular worker’s duties is equivalent to that of a regular employee, their working conditions should be the same. In other words, non-regular workers should be integrated into the same labor management framework as regular employees (Monosashi-ken, 2000; Paato-ken, 2002). The remaining issue, which became central in the subsequent revision of the *Paato* Law, is how to determine when the content of work can be regarded as substantively equivalent.

While the report adopts the principle of “equal pay for equal work,” as is common in many European contexts, it simultaneously argues that the same institutional arrangements cannot be replicated in Japan due to the absence of an occupational labor market grounded in craft unionism and standardized occupational wage structures. Instead, the report frames its discussion around the notion of “non-regular employees who work in the same manner as regular employees,” thereby constructing regular employment as the normative point of reference. Since this logic of “standard employment centrism” (Vosko, 2011) guided the labor market re-regulations implemented in the late 2000s, it placed subsequent reforms on a path-dependent trajectory.

The revision of the *Paato* Law exemplifies this form of re-regulation—one that remains entrenched in, and ultimately affirms, the underlying logic of company citizenship without fundamentally questioning it.

#### 4.2.2 Close enough to regular workers?: the case of *Paato* law

The revision most directly influenced by these reports was the amendment of the *Paato* Law. Originally enacted in 1993, the law aimed to improve the working conditions of part-time workers engaged in jobs comparable to those of regular employees. The three partially overlapping structural shifts discussed earlier heightened the urgency for reform. In response to growing public criticism, the LDP-led government requested that the advisory committee within the MHLW^9^ begin deliberations on addressing the inequality between regular and non-regular employment in July 2006. Shortly thereafter, the Abe administration was inaugurated and designated “equal treatment” as a central policy initiative.

Deliberations within the committee^10^ quickly centered on the fundamental question: *who should be entitled to equal treatment with regular employees?* Labor unions advocated for a standard based on the substantive content of the work performed. Employers, by contrast, contended that since wages in Japan are not strictly determined by job content, pay parity could only be justified when workers perform identical tasks *and* accept equivalent labor management requirements, such as job rotation and geographic transfers (EEC, 2006). As the committee chair underscored the continued relevance of the two earlier reports, discussions remained within the existing conceptual framework and tended to align with employer interests.

Toward the end of 2006, the committee finalized its proposal for the revision of the *Paato* Law. The proposal advanced two major recommendations:

1. The terms of employment contracts should be clearly and explicitly defined, and part-time workers who perform duties equivalent to those of regular employees should be granted equal treatment;
2. Violations of these standards should be subject to legal sanction, rather than relying solely on voluntary efforts by firms.

With respect to the criteria for determining equivalence to regular employees, the committee identified the following three conditions:

1. The scope of job duties and responsibilities is comparable to that of regular employees;
2. The employment relationship is either indefinite or consists of consecutively renewed fixed-term contracts;
3. The worker is subject to the same scope of job rotation and transfers as regular employees.

The committee acknowledged that performing the same tasks alone was insufficient for establishing equivalence. To be considered comparable—and thus eligible for inclusion in the same labor management framework—non-regular workers must also accept a similar range of responsibilities and demonstrate a comparable level of organizational commitment. These criteria were directly incorporated into Article 8 of the revised *Paato* Law, enacted in 2008.

The underlying message is clear: non-regular workers are not entitled to the same treatment as regular employees unless they fulfill the responsibilities associated with corporate flexibility as defined by the logic of company citizenship. While the revised law may contribute to remedying discriminatory treatment in the most evident cases—specifically, *paato* workers whose organizational roles are virtually identical to those of regular employees (a group that comprises only a small fraction of all *paato* workers)—its broader effect is quite different. Rather than challenging the structural division between employment statuses, the reform codifies this distinction in legal terms. In doing so, it formalizes and legitimizes a system of differentiated entitlements to industrial citizenship, effectively reinforcing the exclusion of the vast majority of *paato* workers.

### 4.3 Diversification and stratification of regular employment

While the rise of non-regular employment attracted attention during the 2000s, changes were also occurring within the category of regular employment. One notable development was the expansion of so-called “limited regular employment” (*gentei seishain,* hereafter, LRE). This type of employment includes various forms of restrictions (for employers), such as on work location (area), job contents, or working hours. In fact, a form of LRE was already institutionalized in the 1980s. Its origins can be traced back to the introduction of separate regular employment into career track and clerical track in response to the 1986 Equal Employment Opportunity Law. The clerical track was, in practice, designed for young women and entailed limitations in terms of job content, location, and working hours compared to the career track^11^. Today’s LRE is widely recognized as part of the diversification of working styles within the career track category. For instance, LRE with location limitation (area-type) has become increasingly common. Certain job types, such as sales, are particularly suited to such area-type LRE and are often labeled as “career track (area-based)” on recruitment websites.

Although this form of employment expanded significantly during the 2000s, by the early 2010s the Japanese government began to actively promote it as a policy measure to improve work–life balance, which was framed as a system to support the diversification of working styles. Today, the term *diversity* is frequently invoked in policy and corporate discourse, and when used in reference to work style reform, it often implies the utilization of the LRE*s.* Against the backdrop of an aging population, the working-age generation is increasingly burdened not only with childrearing but also with elder care responsibilities. The structure of care obligations is itself diverse, and the LREs were designed, at least in principle, to accommodate such diversity^12^.

However, we must ask whether this “diversification” genuinely reflects a diversification of working styles alone. If this were the case, such arrangements could be seen as appropriately responding to the needs of workers with diverse circumstances. Yet in practice, this diversification has been accompanied by increasing stratification. For instance, among companies that have adopted the LREs, approximately 50% set the wage levels of such employees at between 80% and less than 100% of their non-limited regular counterparts. Additionally, around 30–40% of these firms offer even lower wage levels, clearly demonstrating that LREs tend to earn less. Moreover, the promotion ceilings for these workers are generally set lower than those for non-limited regular employees. In other words, the resources and opportunities associated with limited regular employment are structurally inferior.

The expansion of LREs has served to stratify regular career track employees based on their ability to meet the expectations of company citizenship. One prominent axis of differentiation is transferability, particularly the geographical scope of job relocation. Whereas regular career track employees were once treated as a uniform category, many firms now distinguish between “national employees,” who are subject to nationwide (and occasionally international) transfers, and “regional” or “area-based employees,” whose transfer obligations are restricted or nonexistent. The latter are typically compensated at 80–90% of the wage level of their national counterparts and face explicit promotion ceilings, reinforcing hierarchical divisions within the regular career track workforce.

**Figure 5:**
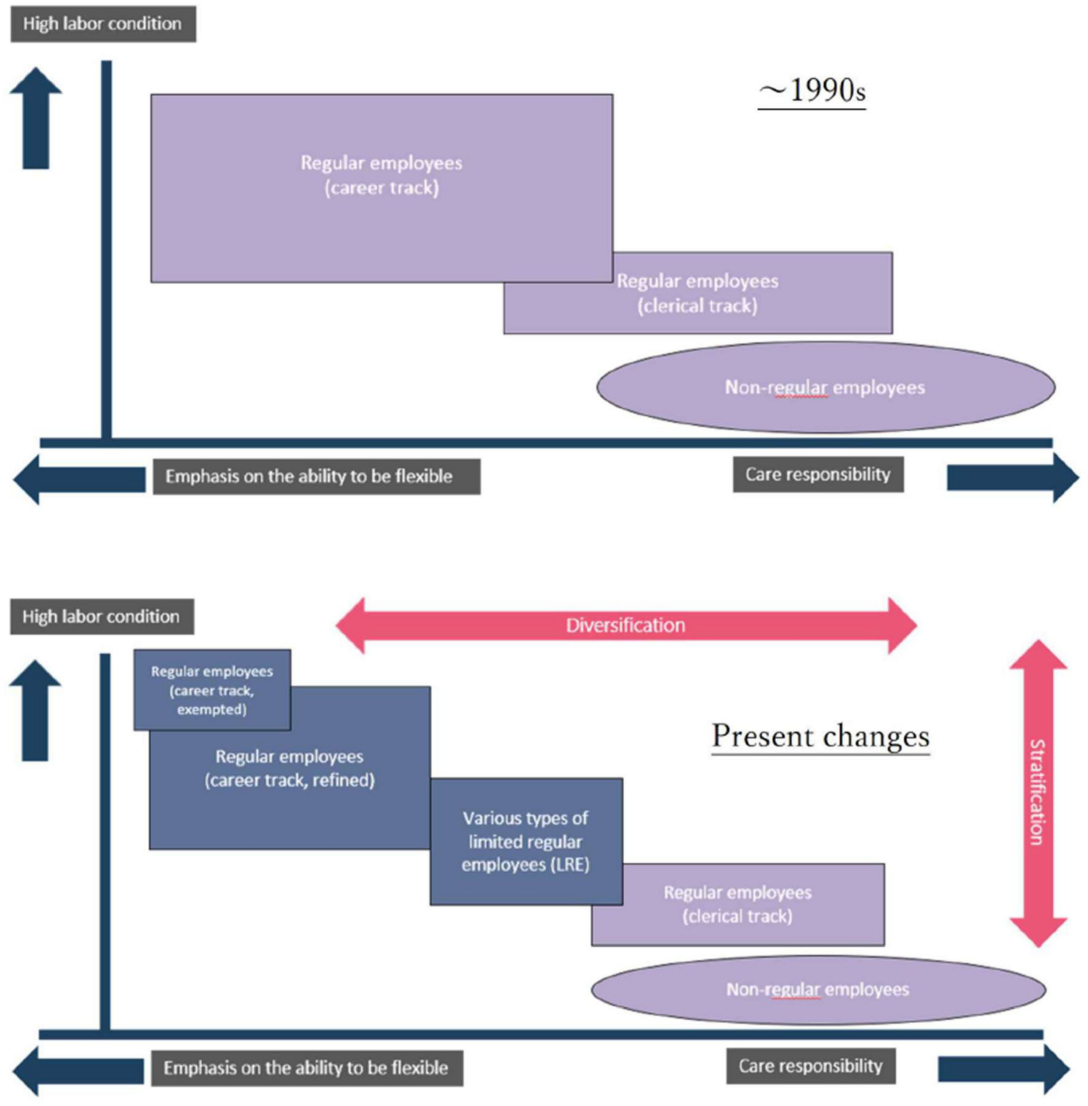
The diversification and stratification of regular employment: from the 1990s to the 2020s (revised and translated from Imai, 2024, p. 114).

In short, while the diversification of working styles in Japanese firms does indeed acknowledge a wider array of employment patterns, the institutional design of these systems remains grounded in the logic of company citizenship. That is, a mechanism of stratification is embedded in which workers who are highly responsive to corporate demands for mobility and flexibility are granted higher compensation, whereas those with caregiving responsibilities—who are less able to meet such expectations—are assigned lower levels of employment benefits and advancement. This dynamic is visually represented the following Figure 5.

The employment structure from the 1980s to the 1990s was characterized by a combination of regular employees – career and clerical tracks – and non-regular employees, as shown in the top panel. In the bottom panel, the category of original regular employees (career track) is depicted as having diversified into multiple categories, including “regular employees (career track, exempted),” “regular employees (career track, refined),” and various types of LREs. Among these, “regular employees (career track, refined)” represents non-limited regular employees, as certain types of LREs are exempt from geographic transfers, effectively shifting the burden of such mobility onto those in higher-tier categories. As a result, refined regular employees and those above are typically compensated at higher levels and afforded greater opportunities for promotion compared to their limited-track counterparts. The same logic applies to the category of “regular employees (career track, exempted).” Although not discussed in this paper, some workers are subject to special working time regulations, similar to the “white-collar exemption” in the United States. These workers are subject to more flexible and often demanding work schedules than even traditional regular employees and therefore enjoy the highest wage levels and the greatest prospects for upward mobility (Imai, 2011a, 2021).

Diversification of employment has proceeded hand in hand with stratification of labor contracts. At the core of this dynamic of stratification lies a shared recognition—by both employers and workers—that the resulting inequalities and exclusions are legitimate, insofar as they are grounded in workers’ willingness and attitude to fulfill the obligations associated with company citizenship. The companies that implemented LREs explained that the reforms were intended to address complaints that it was unfair for transferred and non-transferred employees to receive the same treatment (Imai, 2021, p. 280–283).

## 5 Conclusion: reframing class relations through the lens of citizenship

This paper has argued that the stratification of workers in Japan should be understood through the concept of *industrial citizenship*—a historically constructed set of rights and obligations attached to specific categories of workers, formed through collective negotiations among labor, employers, and the state. This framework reinterprets what are conventionally understood as “class” as the outcomes of contestations over the expansion and institutionalization of citizenship—processes that intertwine notions of contract and status—rather than as expressions of economic struggles. In the Japanese context, prevailing patterns of inequality—differentiated by firm size, gender, and employment status—are not adequately explained by conventional class categories. Rather than attributing this to Japanese exceptionalism, this paper has proposed that it reflects the limited analytical utility of class variables themselves. Inequality should instead be approached as the product of historically situated institutional arrangements, in which class dynamics give rise to distinctive citizenship norms and hierarchies specific to each society.

In Japan, industrial citizenship emerged in the postwar period as *company citizenship*, with regular employment status confined within the organizational boundaries of individual firms. As industrial citizenship developed into company citizenship, the primary lines of inequality and exclusion in Japan came to be defined not by “class,” as commonly emphasized in Western contexts, but by company size, gender, and employment status. Although class dynamics—understood as the negotiation and contestation between workers and employers—were present, the resulting categories and hierarchies diverged significantly from those typically associated with class in Western societies.

The norms of company citizenship has retained its influence in recent decades, even as it became increasingly formalized. Since the oil crisis, and against the backdrop of the establishment of management prerogative, company citizenship in Japan has shifted in emphasis. It now highlights flexible abilities, including a mindset in which workers subordinate their own needs to those of the firm—an orientation regarded as a form of competency. Even during the post-bubble period marked by neoliberal reforms, the expanding authority of employers further advanced the stratification of workers based on the evaluation of such competencies. Yet up to that point, company citizenship, although widely shared in practice, remained an informal norm. The subsequent processes of labor market deregulation and re-regulation, which facilitated the expansion of non-regular employment, can be understood as a redefinition of non-regular workers in relation to regular employees. These reforms clarified the scope and content of workers’ rights and obligations through legal codification.

The legacy of company citizenship in Japan extends beyond the divide between regular and non-regular employment. It also underpins the stratification of regular employees themselves.^13^ In particular, differentiated employment tracks—referred to as LREs—have emerged based on the extent to which workers are expected to meet employers’ demands regarding transfers and working hours. While the creation of an intermediate layer between regular and non-regular employees may, in some cases, appear to mitigate the concentration of wealth, the institutionalization of these separate employment tracks has clearly deepened the segmentation among employees. This segmentation fosters a long-term accumulation of disparities in access to resources and opportunities for mobility. Importantly, this process cannot be attributed solely to managerial prerogative; rather, it unfolds through an individualized process of carefully mobilized worker consent^14^, whereby stratification comes to be perceived as legitimate inequality^15^.

The concept of industrial citizenship provides a useful framework for examining how the dynamics of labor relations generate divergent patterns of social inequality, inclusion, and exclusion. This process enabled the employment contract by incorporating and negotiating status norms specific to the society. The resulting social order is robust and durable. This is, first, because the dynamic process interweaves different levels of negotiation, such as policy and labor-management interactions (Tilly, 1998). It also mobilizes workers’ mindsets and attitudes around hard work and fair competition, thereby reinforcing its legitimacy. This paper uses the case of Japan to demonstrate how these dynamics have shaped such outcomes.

## Data Availability

The original contributions presented in the study are included in the article/supplementary material, further inquiries can be directed to the corresponding author.

## Footnotes

^1^In this respect, stratification research exhibits flexibility in identifying variables of inequality. However, it lacks the ability to account for historical contexts as the class concept does.

^2^All of these publications are based on the author’s original research, including analyses of legislative processes through council minutes and interviews with council members, as well as analyses of labor management practices using materials and interviews provided by specific companies.

^3^Marxist class schemas begin with the antagonistic relationship between labor and capital, both assumed to behave in a utilitarian manner, and have successfully elaborated concepts such as economic and legal ownership and control. These concepts are combined into a matrix of ownership and control, from which notions like “contradictory class locations” are derived (Wright, 1982). The problem, however, lies in their tendency to interpret all changes—including fundamental epistemic shifts in the very concept of “ownership,” such as the historical break with slavery and the recognition of workers as the owners of their own labor power, which made the conflict over ownership possible in the first place—solely through the development of capitalism (Wright, 2005, p. 11). In later work, Wright does come to emphasize the role of power and “rights” in production relations (Wright, 2005). Yet for such schemas to be fully developed, it is essential to extend the analytical horizon beyond capitalist development alone, and to incorporate the historical development of citizenship rights into the discussion.

^4^Japan’s vertical corporate hierarchy, originally established during the wartime, was later reinstated by MITI to enhance competitiveness during the period of rapid economic growth (Lincoln and Gerlach, 2004, p. 75). As labor union activity was confined to individual firms—where company unions aimed to secure better “citizenship” for all employees—this vertical *keiretsu* structure became a key basis for firm-size-based inequality.

^5^The earlier example is reported by Yoshida (2024), He documents the removal of women from the shop floor at Nissan—one of Japan’s largest automakers—and their subsequent reassignment or re-employment as temporary workers following the implementation of the Dodge Line policy in 1949.

^6^While “*paato*” literally translates to “part-time worker,” this paper retains the Japanese term to reflect its context-specific usage. In the Japanese context, “*paato*” often refers to workers whose schedules approximate full-time hours, suggesting that the term primarily signifies a status distinction rather than contractual working time.

^7^See footnote 6 for the definition of “*paato*.” “*Keiyaku*” refers to contract workers, and “*haken*” to temporary agency-dispatched workers. All are categorized as non-regular employees.

^8^Where non-regular work is not equivalent to regular work, the reports call for “balanced consideration” to prevent inequality and exclusion. This principle subsequently underpinned court rulings requiring the payment of various expenses, such as transportation costs, to non-regular employees.

^9^MOL was integrated with the Ministry of Welfare and re-organized as MHLW in 2001.

^10^Equal Employment Commission under the Advisory Council of Labor Policy.

^11^This vertical gender division of labor within the regular employment category is widely recognized as a key factor contributing to gender disparities in Japan, including the low representation of women in managerial positions, among other forms.

^12^It is significant that these tracks have been institutionalized not as temporary statuses but as long-term career paths. While labor unions advocated for treating such positions as provisional and based on temporary circumstances, management asserted the legitimacy of assigning workers to separate tracks from the point of hire, arguing that investing in long-term personnel development was unjustifiable for those not expected to meet the full obligations of standard career trajectories.

^13^Sonoda shows that company citizenship is also reflected in the expectations placed on white-collar foreign workers, and that this has become a factor hindering their full integration (Sonoda, 2023).

^14^As a result, a paradox emerges: citizenship itself becomes individualized. This phenomenon has been characterized by Dukes and Streeck (2023) as the “privatization of citizenship,” a development that may become a focal point for future transformations.

^15^Quantitative analyses have also shown that fulfilling the obligations of company citizenship is recognized by workers themselves as a legitimate basis for wage disparities (Arita et al., 2019).
